# Impact of Excess Adiposity on Cardiorespiratory Fitness in Children and Adolescents with Kawasaki Disease

**DOI:** 10.3390/life15020264

**Published:** 2025-02-10

**Authors:** Guan-Bo Chen, Sheng-Hui Tuan, Yi-Ju Tsai, I-Ching Huang, I-Hsiu Liou, Ko-Long Lin

**Affiliations:** 1 Department of Internal Medicine, Kaohsiung Armed Forces General Hospital, Kaohsiung 802, Taiwan; tanwein@gmail.com; 2 Division of Gastroenterology, Department of Internal Medicine, Tri-Service General Hospital, National Defense Medical Center, Taipei 114, Taiwan; 3 Institute of Allied Health Sciences, College of Medicine, National Cheng Kung University, Tainan 701, Taiwan; 4 Department of Rehabilitation Medicine, Cishan Hospital, Ministry of Health and Welfare, Kaohsiung 842, Taiwan; 5 Department of Physical Medicine and Rehabilitation, School of Medicine, College of Medicine, Kaohsiung Medical University, Kaohsiung 807, Taiwan; 6 Department of Physical Therapy, College of Medicine, National Cheng Kung University, Tainan 701, Taiwan; 7 Department of Physical Medicine and Rehabilitation, Kaohsiung Medical University Hospital, Kaohsiung 807, Taiwan; 8 Department of Physical Medicine and Rehabilitation, Kaohsiung Veterans General Hospital, Kaohsiung 813, Taiwan; 9 Department of Physical Medicine and Rehabilitation, Kaohsiung Municipal Siaogang Hospital, Kaohsiung 812, Taiwan

**Keywords:** cardiopulmonary exercise testing, coronary artery Z score, fat-mass index, Kawasaki disease, peak oxygen consumption

## Abstract

(1) Background: To assess the impact of excessive body fat on cardiorespiratory fitness (CRF) and coronary artery health in children and adolescents following acute Kawasaki disease (KD-CA). (2) Methods: A retrospective study of KD-CA patients (ages 8–16) who completed cardiopulmonary exercise testing (CPET) in the last five years. Participants were classified based on body mass index (BMI) and fat mass index (FMI) into normal and excessive adiposity groups. Coronary artery (CA) Z-scores were calculated using the Lambda-Mu-Sigma method, with peak VO2 Z-scores (peak VO2 Z-score) derived from a database of Hong Kong Chinese children and adolescents. Primary outcomes included peak VO2 Z-score, Max-Z (maximum CA Z-score), anaerobic threshold metabolic equivalent (AT MET), peak MET, and pulse oxygen. Statistical analyses were conducted to evaluate differences across groups. (3) Results: A total of 112 KD-CA patients were included (mean age: 11.71 ± 2.54 years). The mean peak VO2 Z-score was −0.63 ± 0.98. Participants with normal BMI and FMI had significantly higher pulse oxygen levels compared to those with excessive BMI and FMI (both *p* < 0.001). Additionally, those with normal FMI showed higher AT MET, peak MET, peak VO2 Z-score (*p* = 0.049), and lower Max-Z (*p* = 0.026) than excessive FMI participants. Boys, especially those with normal adiposity, had superior AT MET, peak MET, pulse oxygen, and Max-Z compared to girls. (4) Conclusions: Excessive adiposity in KD-CA patients is associated with reduced CRF and elevated Max CA Z-score. These findings highlight the need to monitor body composition to optimize cardiovascular health in this population.

## 1. Introduction

Kawasaki disease (KD) is an acute vasculitis primarily affecting children, which can lead to significant cardiovascular complications, most notably coronary artery aneurysm (CAA) [1]. KD has become the leading cause of acquired heart disease in children in many developed countries, including those in East Asia, where the prevalence is particularly high [2]. While Kawasaki disease is a systemic vasculitis that can affect all blood vessels, its most clinically significant and long-term complications are associated with coronary artery involvement. Despite advancements in early detection and treatment with intravenous immunoglobulins and aspirin, approximately 25% of untreated and 5% of treated children with KD may still develop coronary artery aneurysms (CAAs) [1], with lingering cardiovascular risks that extend into adulthood. The risk of coronary artery disease (CAD) in these patients raises concerns about long-term outcomes, particularly as childhood obesity rates continue to rise [3,4].

Obesity and overweight status are well-established risk factors for cardiovascular disease in the general population [5]. Overweight and obesity are pro-inflammatory states [6], whereas KD is an inflammatory-induced immune vasculitis, mainly involving the coronary arteries. Research has shown that obesity is linked to a pro-inflammatory state, which may worsen the coronary inflammation characteristic of Kawasaki disease [4,7]. Given the pro-inflammatory nature of obesity and its established link to adverse cardiovascular outcomes, it is plausible that obesity in KD patients may exacerbate coronary artery involvement, but this relationship remains underexplored. To the best of our knowledge, prior to this publication, only one study from China had identified obesity as a potential independent risk factor for the development of coronary artery lesions in KD patients [4].

Cardiorespiratory fitness (CRF) is the ability of the cardiovascular and respiratory systems to supply oxygen to the muscles during sustained physical activity. Obesity/overweight and CRF are independent predictors of cardiovascular and all-cause mortality [8]. Obesity and overweight status could lead to poorer cardiopulmonary fitness in children, adolescents [9], and even preschoolers [10]. In previous studies utilizing direct cardiopulmonary exercise testing (CPET), children with a history of Kawasaki disease exhibited comparable CRF but demonstrated lower myocardial flow reserve and higher total coronary resistance than their healthy counterparts [11]. However, adolescents with a history of KD displayed significantly reduced aerobic metabolism capacity and peak exercise load capacity compared to their peers [12]. Our team found that when comparing CRF across different body mass index (BMI) groups, the overall KD group showed no significant differences; however, overweight boys with KD had notably lower peak oxygen consumption (peak VO2) [13]. It’s important to note that BMI encompasses both fat mass and fat-free mass, which may have different relationships with physical fitness and has been shown to correlate poorly with percentage fat mass (%FM) in children and adolescents [14,15]. Fat mass index (FMI) provides a more precise measure of body adiposity by normalizing fat mass to height squared. This distinction is crucial, as studies have shown that FMI is a better predictor of metabolic and cardiovascular outcomes in pediatric populations compared to BMI. Emerging evidence suggests that FMI may more accurately reflect the relationship between body adiposity and CRF in children and adolescents [16,17].

Although measures derived from CPET, such as peak VO2 and peak metabolic equivalent (MET), provide valuable insights into overall cardiovascular health and are considered the gold standard for evaluating CRF, peak VO2—defined as the highest rate at which the body can consume oxygen during maximal or exhaustive exercise—is influenced by factors such as age, maturity, and sex [18]. To account for size-related variations, it should be scaled using the general allometric equation to derive an appropriate size power function, which allows for a more accurate interpretation of physiological changes associated with size [19]. Recent advancements have led to the development of a Z-score system based on allometric scaled peak VO2 values specifically for Southern Chinese children and adolescents (peak VO2 Z-score) [19]. It offers a more refined method for assessing CRF, enabling comparisons across individuals of different sizes and developmental stages. This system offers a more nuanced approach for assessing CRF, facilitating comparisons among individuals of varying sizes and developmental stages. This is particularly crucial in pediatric populations, where growth and development can complicate the interpretation of absolute VO2 values [20].

The coronary artery Z-score system (CA Z-score) offers a standardized approach for assessing coronary artery dimensions in patients with KD [21]. In Taiwan, Lin et al. established reference ranges for coronary artery diameters by evaluating a nationwide cohort of 412 healthy children under the age of 6 years [22]. However, standardized norms for coronary artery measurements in Taiwanese children older than 6 years are currently lacking. To address this gap, Kobayashi et al. introduced the widely recognized coronary artery Z-score equation (ZSP version 4), which utilizes the Lambda-Mu-Sigma method to calculate sex-specific Z-scores for internal coronary artery diameters in children aged 0 to 18.9 years [23]. This equation has demonstrated improved accuracy and reliability compared to earlier regression models [24].

In light of these gaps, the aim of this study is to investigate the impact of excessive adiposity, as measured by FMI, on CRF and coronary artery health in children and adolescents with a history of KD. By classifying KD patients into normal and excessive FMI groups, we seek to compare their CRF using both traditional measures (peak VO2, peak MET) and the Z-score system of peak VO2. Additionally, we aim to assess the differences in coronary artery Z-scores between the normal and excessive FMI groups to determine whether obesity contributes to greater coronary artery involvement in KD patients.

## 2. Materials and Methods

### 2.1. Study Design and Participants

This study was a retrospective cohort analysis carried out at one medical center located in Kaohsiung City, southern Taiwan. We recruited children and adolescents aged 8 to 18 years who were referred from the pediatric cardiology outpatient clinic to the rehabilitation department for regular follow-up of KD between June 2018 and May 2023. The inclusion criteria for participation required patients to have undergone (A) a complete transthoracic echocardiographic examination, (B) a standard 12-lead electrocardiogram, (C) body composition analysis, and (D) a symptom-limited treadmill exercise test. Patients were excluded from the study if they had (A) significant structural heart disease, (B) moderate to severe cardiac valvular disease, (C) significant arrhythmias, (D) ventricular hypertrophy, or (E) any concurrent pulmonary condition or other diseases that could influence cardiopulmonary function. We recorded basic demographic characteristics such as sex, age, body weight, height, and body fat. The study was conducted in accordance with the principles outlined in the Helsinki Declaration and received approval from the Institutional Review Board of Kaohsiung Veterans General Hospital (approval number: VGHKS17-CT11-11). Additionally, this research adhered to the STrengthening the Reporting of OBservational studies in Epidemiology (STROBE) guidelines for reporting.

**Figure 1:**
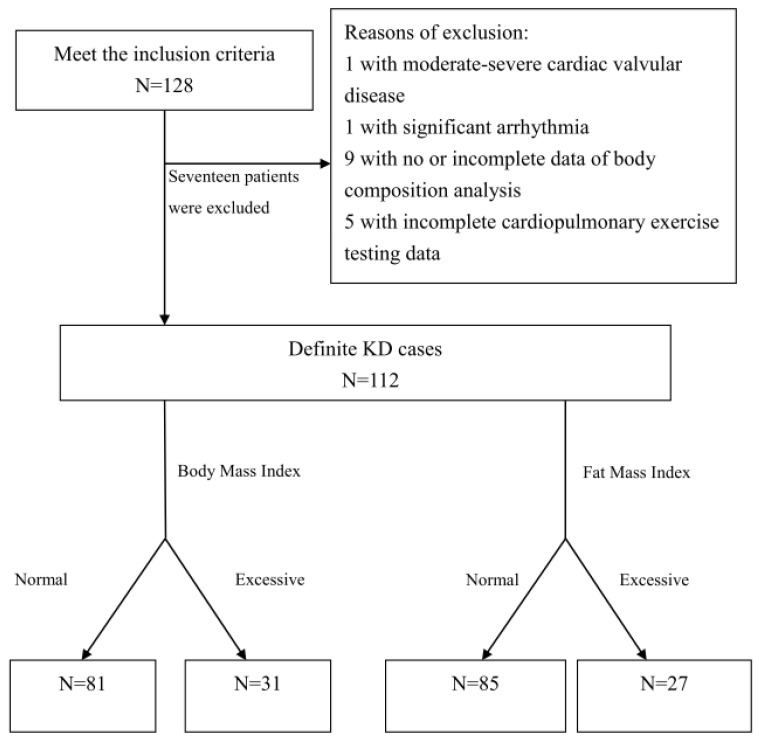
Inclusion Algorithm.

One hundred twenty-eight patients with KD qualified for the inclusion criteria. Of these, sixteen patients were excluded (one each with moderate and severe cardiac valvular disease, one with significant arrhythmia, nine with no or incomplete data of body composition analysis, and five with incomplete CPET data). Therefore, 112 children and adolescents with KD were included in this study (Figure 1).

### 2.2. Anthropometry-Body Composition

Height was measured using a standardized stadiometer (DETECTO, Model 3PHTROD-WM, Webb City, MO, USA), with participants standing barefoot, ensuring proper posture with their backs straight and heels together. Body composition, including percentage of body fat, total body water, appendicular skeletal muscle mass, and bone mass, was assessed using the Zeus 9.9 PLUS analyzer (Jawon Medical Co., Ltd., Kungsang Bukdo, Seoul, Republic of Korea), which employs the tetrapolar electrode method through vector bioelectrical impedance analysis. The analyzer automatically calculated BMI by dividing body weight (kg) by the square of height (m^2^). FMI was manually calculated as fat mass (kg) divided by height squared (m^2^). Participants were subsequently classified into normal and excessive adipose groups based on both BMI and FMI criteria. For the BMI classification, participants were grouped according to the age- and gender-specific BMI reference values recommended by the Ministry of Health and Welfare in Taiwan [25]. According to this guideline, overweight is defined as a BMI above the 85th percentile, and obesity is defined as a BMI above the 95th percentile for a given age and sex [25]. Those with a normal BMI were categorized as the normal adipose group, while those identified as overweight or obese were classified into the excessive adipose group. In terms of FMI classification, excess adiposity was defined as an FMI greater than the 75th percentile for each sex, as suggested by Weber et al. [26], while all others were categorized as having normal FMI.

### 2.3. Cardiopulmonary Exercise Testing and Equation of Peak Oxygen Consumption Z-Score

All participants in this study underwent symptom-limited exercise testing using a treadmill, a flow module, a gas analyzer, and an electrocardiographic monitor (Metamax 3B; Cortex Biophysik GmbH Co., Leipzig, Germany). Data were recorded at 30-s intervals to effectively capture physiological changes during exercise. Prior to the CPET, each participant was acquainted with the procedures and testing equipment through a demonstration conducted by an experienced physical therapist. The CPET was conducted using the Bruce ramp protocol, as recommended by the American College of Sports Medicine (ACSM), due to its particular advantages for children and adolescents. This protocol is specifically designed to accommodate varying levels of physical fitness, providing a gradual and progressive increase in workload, which allows for a more accurate assessment of cardiopulmonary function across different fitness levels [27]. The test was terminated once participants reached maximal effort, as defined by the American College of Sports Medicine (ACSM) criteria, which include a respiratory exchange ratio (RER) ≥ 1.1, the presence of a VO2 plateau, and achieving more than 85% of the maximal estimated heart rate (HR). The test was also stopped if participants exhibited unbearable subjective symptoms or were unable to continue [27]. A physiatrist with over 20 years of experience in CPET (K.-L.L.) supervised the entire testing process.

VO2 and carbon dioxide production (VCO2) were measured during the test using the breath-by-breath method. Blood pressure (BP), HR, and RER were continuously monitored throughout the procedure. The peak rate pressure product (PRPP)—an indicator of myocardial oxygen demand and workload during exercise—was calculated by multiplying peak systolic BP by peak HR [28]. The anaerobic threshold (AT) was assessed using the VE/VO2 and VE/VCO2 methods [29]. The VO2 at the anaerobic threshold (AT VO2) and the maximum oxygen uptake recorded at peak exercise (peak VO2) were both determined by the same physiatrist (K.-L.L.). To calculate metabolic equivalent (MET), the measured VO2 was divided by a constant of 3.5 mL/kg/min. The pulse oxygen was defined as peak VO2 divided by peak HR. The peak VO2 Z-score was determined using the scaling equation proposed by Yu et al., which is suitable for Chinese children and adolescents, taking into account age and sex [19]. The peak VO2 Z-score can be obtained via an automated Excel file (https://www.ncbi.nlm.nih.gov/pmc/articles/PMC6413916/, accessed on 6 January 2024).

### 2.4. Echocardiography and Coronary Artery Z-Score

Two experienced pediatric cardiologists conducted examinations while subjects were positioned supine or in the left lateral decubitus position, utilizing two-dimensional, M-mode, and Doppler echocardiography with a sector probe of greater than 5 MHz frequency. Coronary artery luminal diameters were measured from inner edge to inner edge across all segments, avoiding branching points. Measurements for the right coronary artery (RCA) and left anterior descending coronary artery (LCA) were taken 3–5 mm distal to their origins in the parasternal short-axis view. The whole echocardiography was performed following the AHA scientific statement on KD [1].

Coronary artery luminal dimensions were evaluated using the Z-score method as outlined by Kobayashi et al. [23]. The CA Z-score was calculated by entering sex-specific data on age, body height, body weight, body surface area (BSA) using the Haycock formula, and the CA diameter obtained from echocardiography into the ZSP version 4 calculator. The maximum Z-score from either the proximal LCA or RCA was termed Max-Z.

### 2.5. Statistical Analysis

All analyses were conducted using SPSS for Windows version 21.0 (IBM Corp., Armonk, NY, USA). Continuous data are reported as mean ± standard deviation. Normality and homoscedasticity were assessed prior to each analysis. To compare data between sexes and among different BMI and FMI groups, an independent *t*-test was employed for normally distributed variables, whereas the Mann-Whitney U test was utilized for non-normally distributed variables. A *p*-value of less than 0.05 was deemed statistically significant.

## 3. Results

### 3.1. Study Population

The intervals between the initial diagnosis of KD and CPET, and between echocardiography and CPET for our participants were 9.51 ± 5.24 days and 5.30 ± 1.62 days, respectively. Table 1 presents the baseline characteristics of the participants. A total of 112 children were included in the study, with a mean age of 11.71 ± 2.54 years. The age distribution for boys (8 to 16 years) was 7, 8, 9, 4, 9, 9, 5, 3, and 7 participants, respectively. For girls (8 to 18 years), the distribution was: 6, 6, 9, 4, 4, 6, 6, 3, 5, 1, and 1 participant(s), respectively. The mean BMI for the entire cohort was 20.09 ± 4.16 kg/m^2^. When categorized by sex, boys (*n* = 61) had a mean age of 11.7 ± 2.54 years and a BMI of 20.48 ± 4.24 kg/m^2^. In contrast, girls (*n* = 51) had a mean age of 11.73 ± 2.56 years, and a BMI of 19.63 ± 4.07 kg/m^2^. No significant differences in baseline characteristics were observed between boys and girls, except for body fat measurements. Boys exhibited significantly lower percentages of body fat (*p* < 0.001), FMI (*p* = 0.018), FFM (*p* = 0.001), and FFMI (*p* < 0.001) compared to girls.

### 3.2. Comparisons of Cardiopulmonary Fitness Between Participants with Normal and Excessive Body Adipose by BMI and FMI Classification

Table 2 presents the comparisons of CRF among participants categorized by BMI and FMI. The overall cohort comprised 81 participants with normal BMI and 31 with excessive BMI, as well as 85 participants with normal FMI and 27 with excessive FMI. Each group included normal BMI patients (40 boys and 41 girls) compared to those with excessive BMI (21 boys and 10 girls) and normal FMI patients (46 boys and 38 girls) compared to those with excessive FMI (15 boys and 13 girls). Regardless of BMI or FMI subgroups, the mean peak RER values for both the normal and excessive groups exceeded 1.1 (*p* = 0.914 for the BMI group and *p* = 0.637 for the FMI group), indicating that maximal oxygen exercise efforts were achieved.

**Figure 2:**
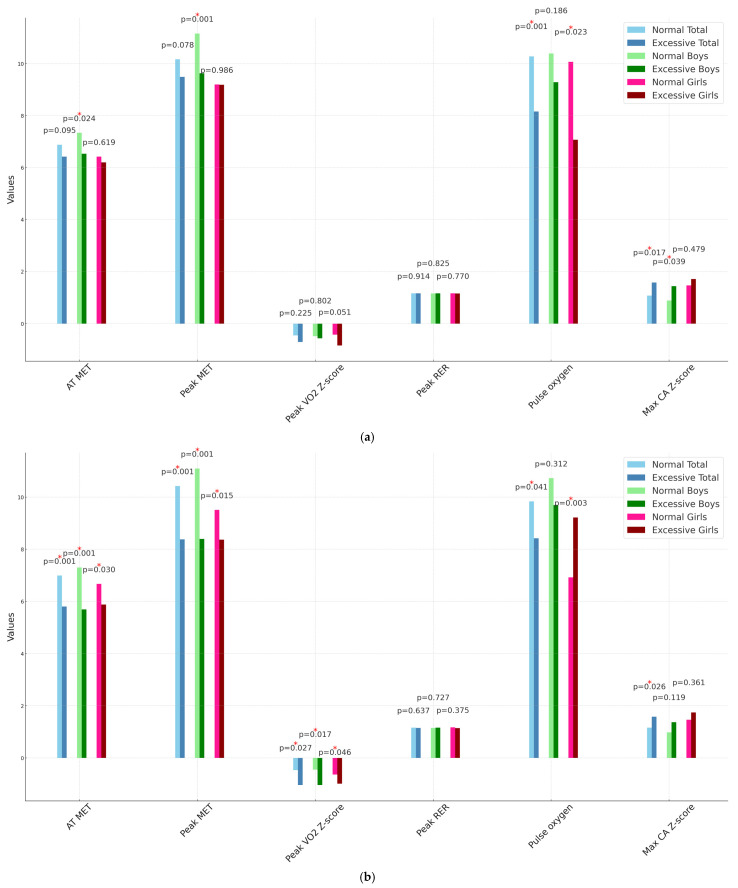
(**a**) Comparisons of cardiopulmonary fitness between participants with normal and excessive body adipose by body mass index (**b**) Comparisons of cardiopulmonary fitness between participants with normal and excessive body adipose by fat mass index. AT MET (unit: MET), metabolic equivalent at the anaerobic threshold; Peak MET (unit: MET), peak metabolic equivalent during exercise testing; Peak VO2 Z-score (unit: Z score), Z-score of peak oxygen consumption based on reference value [19]; RER, respiratory exchange ratio; Max CA Z-score (Unit: Z score), the maximum coronary artery Z-score of the right coronary artery and left anterior descending coronary artery based on Lambda-Mu-Sigma method [23]; * *p* value < 0.05; Note: PRPP was not presented since its scale is significantly larger than that of the other variables.

In terms of BMI classification, normal BMI participants (*n* = 81) had significantly higher pulse oxygen levels (*p* = 0.001), along with a lower Max CA Z-score (*p* = 0.017) compared to those with excessive BMI (*n* = 31). Boys with normal BMI (*n* = 40) showed significantly higher values for AT MET (*p* = 0.024), peak MET (*p* = 0.001), as well as a lower Max-Z (*p* = 0.039) compared to boys with excessive BMI (*n* = 21). Girls with normal BMI (*n* = 41) had only significantly higher pulse oxygen levels (*p* = 0.023) compared to girls with excessive BMI (*n* = 10) (Figure 2a).

In the FMI analysis, normal FMI participants (*n* = 85) had significantly higher values for AT MET, peak MET (both *p* < 0.001), peak VO2 Z-score (*p* = 0.027), and pulse oxygen levels (*p* = 0.041), along with a lower Max-Z (*p* = 0.026) compared to those with excessive FMI (*n* = 27). Boys with normal FMI (*n* = 46) demonstrated significantly higher AT MET, peak MET (both *p* < 0.001), as well as a higher peak VO2 Z-score (*p* = 0.017) compared to boys with excessive FMI (*n* = 15). Girls with normal FMI (*n* = 38) exhibited significantly higher values for AT MET (*p* = 0.030), peak MET (*p* = 0.015), peak VO2 Z-score (*p* = 0.046), and pulse oxygen levels (*p* = 0.003) compared to girls with excessive FMI (*n* = 13) (Figure 2b).

### 3.3. Comparison of Cardiorespiratory Fitness Between Sexes with Normal and Excessive BMI or FMI

Table 3 summarizes the comparisons of CRF between boys and girls with normal and excessive body adiposity, categorized by BMI and FMI. Among the overall cohort, boys exhibited significantly higher AT MET (*p* = 0.005), peak MET, and pulse oxygen levels (both *p* < 0.001) while showing lower Max-Z (*p* = 0.027) compared to girls.

When examining the normal BMI and FMI subgroups, boys consistently outperformed girls in both AT MET and peak MET, with *p*-values ranging from <0.001 to 0.012. Boys with normal BMI and FMI also had significantly lower Max-Z compared to girls (*p* = 0.002 for BMI and 0.012 for FMI). Additionally, boys with normal FMI exhibited higher PRPP (*p* = 0.011) than girls. Notably, in the excessive BMI and FMI groups, boys had only higher pulse oxygen levels (*p* = 0.025 for BMI and 0.005 for FMI) compared to girls. Conversely, girls with excessive body adiposity demonstrated higher AT MET (*p* = 0.022) and peak MET (*p* = 0.036).

## 4. Discussion

To the best of our knowledge, this is the first study to comprehensively evaluate CRF in children and adolescents with KD by comparing peak VO2 Z-scores across different BMI and FMI classifications, as well as between sexes. The primary objective of this study was to investigate the impact of excessive adiposity on CRF and coronary artery involvement in KD patients. Our findings indicate that after the acute stage of KD, while most children and adolescents were able to exert sufficient effort to achieve peak performance during CPET, they still exhibited mildly decreased peak VO2 Z-scores compared to normal reference values. Additionally, KD children and adolescents with excessive FMI specifically had lower AT MET, peak MET, and peak VO2 Z-scores, along with higher Max-Z values—a trend not observed in the BMI classification. This emphasizes the importance of using FMI over BMI in assessing body composition-related impacts on CRF in KD patients.

Many previous studies examining exercise performance and aerobic capacity in children and adolescents with KD have found no significant differences in aerobic metabolism and peak exercise load capacities compared to control groups [11,30,31], despite some ongoing debate in the literature. However, it is important to note that healthy controls in these studies were often drawn from existing databases of normal participants, matched by sex, age, and either BMI or BSA. This matching process may have introduced potential selection and population biases that could affect the study results. In our study, the mean peak VO2 Z-score for all KD children and adolescents was −0.63 ± 0.98. The peak VO2 Z-score represents the deviation of the measured peak MET from the predicted peak MET, using age- and sex-specific references established from a large cohort of Southern Chinese children and adolescents [19]. This suggests that our findings may provide a more accurate reflection of the exercise capacity in this population compared to prior research. We observed that KD children and adolescents had lower peak MET values than their healthy counterparts. Consistent with our results, a recent study by Yang et al. reported that adolescents with a history of KD had significantly lower peak VO2/kg (approximately 7.93%) than their controls [12]. Furthermore, the condition of the coronary artery may influence the results of CPET in KD patients. Our findings indicated that children with KD who had a higher Max CA Z-score exhibited significantly lower peak exercise capacity, regardless of whether measured by peak MET [32] or peak VO2 Z-score [33]. Gravel et al. found that the presence of CAAs in the subacute phase of KD does not affect exercise parameters, but exercise-induced myocardial perfusion defects later correlate with abnormal recovery parameters [34]. The discrepancies between our findings and those of previous studies could stem from variations in coronary artery status among the participants included. It would be beneficial to compare the distribution of CA Z-scores across these studies; however, this is challenging since many prior studies did not report coronary artery status or Z-scores.

Research from various countries has shown that children and adolescents with excessive body fat (overweight or obese) exhibit lower CRF compared to those with normal body fat levels [35,36]. Our team also identified a strong negative correlation between CRF levels and BMI in preschoolers [10] and children [9] in Taiwan. Studies specifically examining CRF among children and adolescents with KD categorized by different BMI or FMI are limited [4,13]. Our findings align with previous research in general. Notably, we found that across all participants and within each subgroup of boys and girls, those classified with excessive body fat based on FMI had poorer peak MET and peak VO2 Z-scores compared to their peers with normal body fat. However, this significant difference was not observed when using BMI as the classification criterion. This discrepancy may arise from the superior effectiveness of fat mass FMI in assessing obesity compared to BMI in children and adolescents. A systematic review and meta-analysis revealed that BMI lacks sensitivity and fails to identify more than 25% of children aged 4 to 18 years with excessive body fat [37]. While obesity is defined as excess body fat, BMI measures only body weight without considering composition; therefore, increases in BMI may not accurately reflect actual rises in body fat percentage, as research suggests that adiposity could increase more significantly than indicated by BMI [17].

In addition to the significant differences in CRF, we found that children and adolescents with KD who had excessive body adiposity (as defined by both BMI and FMI) exhibited higher Max-Z scores. The CA Z-score system measures how many standard deviations a given measurement is above or below the age- and size-specific population mean, allowing for more effective discrimination of CAAs and providing better correlations with clinical outcomes [11]. KD patients with CAAs or higher Max-Z scores tend to have poorer CRF [32,33] and cardiovascular outcomes [38,39]. A baseline Max-Z score of ≥2.0 in children with KD has shown high predictive value for the later development of CAAs [40]. In our study, the mean Max-Z score for participants with excessive adiposity was 1.57 ± 1.02 in the BMI group and 1.71 ± 1.03 in the FMI group, both of which were below the threshold of 2.0. Although small CAAs can regress to normal lumen diameters, this regression may still be associated with abnormalities in vessel wall reactivity, intimal thickness, and cardiovascular events later in life [41]. Notably, while current studies have not specifically evaluated the relationship between CA Z-scores and body fat, overweight and obesity have been independently linked to an increased likelihood of coronary artery lesions among KD patients [4]. Obesity also affects the clinical course of multisystem inflammatory syndrome in children associated with COVID-19. Apart from coronary artery lesions, obesity was associated with worsened inflammatory markers [42]. Excessive body adiposity may serve as a risk factor for coronary artery disease as it relates to energy balance, inflammatory responses, and immune function [43]. Additionally, body fat is associated with coronary vasoreactivity, and reduced vasoreactivity is among the earliest signs of developing coronary artery disease [44]. A recent study showed that adolescents with obesity exhibit increased inflammation, characterized by heightened monocyte activation, elevated cytokine production, and upregulated inflammatory pathways. This chronic inflammatory state is associated with worsened subclinical cardiovascular outcomes, suggesting the need for adjunctive anti-inflammatory interventions [45]. Given that KD is an acquired vasculitis, it may exacerbate the impact of excessive body adiposity on coronary artery lesions in these patients. However, it remains unclear whether excessive body adiposity leads to coronary artery lesions and subsequently poorer CRF, if the presence of coronary artery lesions contributes to poorer CRF and the development of excessive body adiposity, or if both factors coexist and interact. The causal relationship requires further investigation in future longitudinal studies with extended follow-up periods.

The NOODLE study underscores the importance of using accurate, population-specific prediction models for CRF assessment, particularly in specific populations such as endurance athletes (EA). Their recalibration of the FRIEND equation for EA highlighted the need for tailored adjustments, as the original model underestimated peak VO2 [46]. This aligns with our study’s approach of utilizing peak VO2 Z-scores to standardize CRF assessment in children and adolescents with KD, ensuring more precise evaluations accounting for age, sex, and body size.

As previous studies have indicated, our findings show that boys had significantly higher fat-free mass, FFMI, AT MET, and peak MET than girls [47,48]. It is expected that boys also exhibited significantly higher pulse oxygen levels, as this metric is calculated by dividing peak VO2 by peak heart rate [49]. These differences in body composition and CRF may result from variations in physiological characteristics, the timing of puberty, and other developmental factors during adolescence [50]. For instance, boys generally experience a later onset of puberty, which allows for a longer period of growth in FFM and contributes to higher CRF levels. Additionally, hormonal differences, such as higher testosterone levels in boys, promote greater muscle mass development, while higher estrogen levels in girls contribute to increased fat deposition. These hormonal influences lead to differences in body composition and cardiovascular responses during exercise [51]. Additionally, behavioral differences and environmental risk factors between boys and girls may influence the relationship between CRF and childhood obesity. For example, boys often engage in higher levels of physical activity, which can enhance CRF and modulate the impact of adiposity on cardiovascular health. Conversely, girls may experience societal pressures that discourage participation in certain physical activities, potentially leading to lower CRF and higher adiposity [48]. The NOODLE study found significant differences in ventilatory efficiency across sexes and age groups among EAs, emphasizing the variability in physiological responses to exercise. Similarly, our study revealed sex-related differences in CRF outcomes, with boys exhibiting higher fat-free mass and better exercise capacity compared to girls [52].

Our results indicated that boys had a significantly lower Max Z score than girls. Hoang et al. reported that among acute KD patients, males exhibited a higher percentage of coronary artery dilation and aneurysms [53]. In our study, the average time from KD onset to echocardiography was 9.51 years, with participants recruited after the acute phase of KD. Miura et al. found that male sex had a hazard ratio of 2.8 for cardiovascular events in their longitudinal study with an average follow-up of 6.4 years [38]. In contrast, Sadeghi et al. reported no significant gender differences in the incidence of cardiac involvement over a 10-year cross-sectional study [54]. The question of whether there are sex differences in coronary artery involvement among KD patients remains a topic of controversy.

Several limitations of this study warrant consideration. First, participants were recruited from a single medical center in southern Taiwan, which may limit the generalizability of our findings to broader populations with different demographic, genetic, and environmental factors. The prevalence of overweight and obesity in our study (27.7%) was higher than that reported in a study from China (18.5%) [4] and the International Kawasaki Disease Registry, which includes data from eight countries across Europe and North America (23.0%) [42]. The characteristics of our study population, including lifestyle habits, healthcare accessibility, and ethnicity, may differ from those of children in other regions or countries, potentially influencing the observed relationships between adiposity and CRF. Additionally, the lack of external control groups, such as healthy children without Kawasaki disease or those from different geographic locations, limits our ability to draw more definitive conclusions about the broader applicability of our findings. A larger, multi-center, or cross-national study with well-matched external controls is needed to further validate our results and establish their relevance in more diverse populations. Second, this study was conducted retrospectively. Although the interval between echocardiography and CPET was less than 1 week, variability in follow-up timing may have affected the results. Additionally, due to the retrospective nature of the study, data on potential confounders such as socioeconomic status, dietary habits, and physical activity levels were not available. As a result, we were unable to perform multivariable regression analysis to further enhance the robustness of our findings. Third, the ZSP version 4 equation, used to calculate CA Z-scores, is based on Japanese data. Due to the current lack of an established CA Z-score equation specific to Taiwanese children over 6 years old, ZSP version 4 remains the most suitable option; however, it may not fully reflect the coronary artery status of Taiwanese KD children and adolescents. Lastly, although the peak VO2 Z-score equation used in this study is based on Southern Chinese pediatric data, potential ethnic and physiological differences between Taiwanese and Cantonese populations may affect its applicability.

## 5. Conclusions

Our study highlights the significant impact of body composition, specifically FMI, on CRF and coronary artery involvement in children and adolescents with KD. Our findings demonstrate that KD patients with excessive body adiposity exhibit poorer peak exercise capacity and altered cardiovascular metrics compared to those with normal body fat, emphasizing the need for careful monitoring of body composition and health promotion in this population. The use of peak VO2 Z-scores offers a refined approach for assessing CRF that accounts for variations in age, sex, and body size, allowing for more accurate evaluations of cardiovascular health in KD patients. Clinically, incorporating CRF assessments into routine follow-ups could aid in the early detection and management of at-risk individuals through personalized exercise prescriptions and lifestyle modifications. Furthermore, our research underscores the necessity of further investigations to clarify the causal relationships between excessive body fat, coronary artery lesions, and CRF in KD patients. Given the increasing prevalence of obesity in children, our study advocates for the implementation of targeted weight management strategies and public health initiatives to improve long-term cardiovascular outcomes in this population.

## Figures and Tables

**Table 1:** Baseline characteristics of recruited participants.

	Total (*n* = 112)	Boy (*n* = 61)	Girl (*n* = 51)	*p* Value Between Sexes ^a^
Age (year)	11.71 ± 2.54	11.7 ± 2.54 (11.06, 12.34)	11.73 ± 2.56 (11.03, 12.43)	0.966
Height (cm)	148.57 ± 16.26	150.38 ± 17.17 (146.07, 154.69)	146.42 ± 14.98 (142.31, 150.53)	0.201
Weight (kg)	45.72 ± 16.44	47.45 ± 17.34 (43.10, 51.80)	43.65 ± 15.21 (39.48, 47.82)	0.225
BMI (kg/m^2^)	20.09 ± 4.16	20.48 ± 4.24 (19.42, 21.54)	19.63 ± 4.07 (18.51, 20.75)	0.283
Body fat(%)	19.17 ± 8.20	15.75 ± 8.14 (13.71, 17.79)	23.07 ± 6.36 (21.32, 24.82)	<0.001 *
FM (kg)	9.65 ± 7.14	8.71 ± 7.52 (6.82, 10.60)	10.74 ± 6.60 (8.93, 12.55)	0.143
FMI (kg/m^2^)	4.14 ± 2.59	3.59 ± 2.67 (2.92, 4.26)	4.77 ± 2.37 (4.12, 5.42)	0.018 *
FFM (kg)	36.79 ± 10.80	39.99 ± 10.83 (37.27, 42.71)	33.14 ± 9.62 (30.50, 35.78)	0.001 *
FFMI (kg/m^2^)	16.10 ± 2.33	17.05 ± 2.18 (16.50, 17.60)	15.01 ± 2.00 (14.46, 15.56)	<0.001 *
Excess Adiposity by FMI	24.11%	24.60%	25.49%	
KD duration	9.51 ± 5.24	9.26 ± 4.06 (8.24, 10.28)	10.01 ± 5.60 (8.47, 11.55)	0.414

Notes: data are presented as mean ± standard deviation (95% confidence interval) or percentage; *n*: number; BMI: body mass index; FM: fat mass; FMI: fat mass index; FFM: fat-free mass; FFMI: fat-free mass index; Excess adiposity by FMI: >75th percentile of FIM of each sex as per the suggestion of Weber D.R. et al. [26], KD duration, interval from the first diagnosis of Kawasaki disease to the cardiopulmonary exercise testing; ^a^ All the comparisons between girls and boys were performed by independent *t*-test except *p*-values marked with * *p*-value < 0.05.

**Table 2:** Comparisons of cardiopulmonary fitness between participants with normal and excessive body adipose.

	AT MET	Peak MET	Peak VO2 Z-Score	Peak RER	PRPP	Pulse Oxygen	Max CA Z-Score
BMI Group							
Total (*N* = 112)	6.75 ± 1.29	9.98 ± 1.82	−0.63 ± 0.98	1.16 ± 0.10	28,702.16 ± 6005.15	8.75 ± 3.04	1.43 ± 1.00
Normal (*N* = 81)	6.88 ± 1.31 (6.59, 7.17)	10.17 ± 1.90 (9.76, 10.58)	−0.45 ± 1.16 (−0.70, −0.20)	1.16 ± 0.10 (1.14, 1.18)	30,268.03 ± 6157.08 (28,927.15, 31,608.91)	10.28 ± 3.09 (9.61, 10.95)	1.07 ± 0.87 (0.88, 1.20)
Excessive(*N* = 31)	6.42 ± 1.21 (5.99, 6.85)	9.49 ± 1.55 (8.94, 10.04)	−0.70 ± 0.91 (−1.02, −0.38)	1.16 ± 0.09 (1.13, 1.19)	28,102.88 ± 5874.17 (26,035.02, 30,170.74)	8.16 ± 2.82 (7.17, 9.15)	1.57 ± 1.02 (1.21, 1.93)
*p*-value	0.095	0.078	0.225	0.914	0.088	0.001 *	0.017 *
Boys (*N* = 61)	7.06 ± 1.25	10.63 ± 1.76	−0.53 ± 1.08	1.15 ± 0.08	29,627.82 ± 6018.30	9.67 ± 3.07	1.24 ± 0.99
Normal (*N* = 40)	7.34 ± 1.09 (7.00, 7.68)	11.16 ± 1.54 (10.68, 11.64)	−0.48 ± 1.31 (−0.89, −0.07)	1.15 ± 0.08 (1.13, 1.17)	30,351.62 ± 6397.68 (28,368.96, 32,334.28)	10.39 ± 2.99 (9.46, 11.32)	0.88 ± 0.86 (0.61, 1.13)
Excessive (*N* = 21)	6.53 ± 1.38 (5.94, 7.12)	9.63 ± 1.74 (8.89, 10.37)	−0.56 ± 0.95 (−0.97, −0.15)	1.16 ± 0.09 (1.12, 1.20)	29,247.82 ± 5856.87 (26,742.80, 31,752.84)	9.29 ± 3.07 (7.98, 10.60)	1.43 ± 1.00 (1.15, 1.86)
*p*-value	0.024 *	0.001 *	0.802	0.825	0.501	0.186	0.039 *
Girls (*N* = 51)	6.38 ± 1.26	9.20 ± 1.60	−0.75 ± 0.66	1.16 ± 0.11	27,595.01 ± 5856.42	7.65 ± 2.63	1.66 ± 0.98
Normal (*N* = 41)	6.42 ± 1.36 (6.00, 6.84)	9.20 ± 1.71 (8.68, 9.72)	−0.42 ± 0.52 (−0.58, −0.26)	1.16 ± 0.12 (1.12, 1.20)	30,092.50 ± 5946.32 (28,272.33, 31,912.67)	10.07 ± 3.43 (9.02, 11.12)	1.46 ± 0.79 (1.22, 1.70)
Excessive (*N* = 10)	6.20 ± 0.75 (5.74, 6.66)	9.19 ± 1.06 (8.53, 9.85)	−0.84 ± 0.85 (−1.37, −0.31)	1.15 ± 0.08 (1.10, 1.20)	26,985.85 ± 5742.53 (23,426.59, 30,545.11)	7.07 ± 2.05 (5.80, 8.34)	1.71 ± 1.03 (1.07, 2.35)
*p*-value	0.619	0.986	0.051	0.770	0.134	0.023 *	0.479
FMI Group							
Total (*N* = 112)	6.75 ± 1.29	9.98 ± 1.82	−0.63 ± 0.98	1.16 ± 0.10	28,702.16 ± 6005.15	8.75 ± 3.04	1.43 ± 1.00
Normal (*N* = 85)	6.99 ± 1.24 (6.73, 7.25)	10.43 ± 1.69 (10.07, 10.79)	−0.47 ± 0.75 (−0.64, −0.31)	1.16 ± 0.09 (1.14, 1.18)	28,887.81 ± 6501.77 (27,505.59, 30,270.03)	9.84 ± 3.16 (9.17, 10.51)	1.16 ± 0.77 (1.00, 1.32)
Excessive (*N* = 27)	5.80 ± 0.99 (5.43, 6.17)	8.38 ± 1.24 (7.91, 8.85)	−1.04 ± 1.08 (−1.45, −0.63)	1.15 ± 0.11 (1.11, 1.19)	28,492.94 ± 5673.26 (26,352.97, 30,632.91)	8.42 ± 2.95 (7.31, 9.13)	1.58 ± 1.04 (1.19, 1.97)
*p*-value	<0.001 *	<0.001 *	0.027 *	0.637	0.764	0.041 *	0.026 *
Boys (*N* = 61)	7.06 ± 1.25	10.63 ± 1.76	−0.53 ± 1.08	1.15 ± 0.08	29,627.82 ± 6018.30	9.67 ± 3.07	1.24 ± 0.99
Normal (*N* = 46)	7.30 ± 1.11 (6.98, 7.62)	11.10 ± 1.49 (10.67, 11.53)	−0.45 ± 0.58 (−0.62, −0.28)	1.15 ± 0.09 (1.12, 1.18)	31,532.55 ± 6760.50 (29,578.86, 33,486.24)	10.73 ± 3.42 (9.74, 11.72)	0.98 ± 0.75 (0.76, 1.20)
Excessive (*N* = 15)	5.69 ± 0.98 (5.19, 6.19)	8.40 ± 0.99 (7.90, 8.90)	−1.04 ± 1.30 (−1.70, −0.68)	1.16 ± 0.09 (1.11, 1.21)	28,910.07 ± 5452.82 (26,150.56, 31,669.58)	9.70 ± 2.92 (8.22, 11.18)	1.37 ± 1.04 (0.84, 1.90)
*p*-value	<0.001 *	<0.001 *	0.017 *	0.727	0.177	0.312	0.119
Girls (*N* = 51)	6.38 ± 1.26	9.20 ± 1.60	−0.75 ± 0.66	1.16 ± 0.11	27,595.01 ± 5856.42	7.65 ± 2.63	1.66 ± 0.98
Normal (*N* = 38)	6.67 ± 1.12 (6.31, 7.03)	9.51 ± 1.52 (9.03, 9.99)	−0.64 ± 0.49 (−0.72, −0.58)	1.17 ± 0.10 (1.14, 1.20)	27,928.59 ± 5994.58 (26,022.59, 29,834.59)	6.92 ± 1.82 (6.34, 7.50)	1.46 ± 0.92 (1.17, 1.75)
Excessive (*N* = 13)	5.88 ± 1.03 (5.32, 6.24)	8.37 ± 1.42 (7.60, 9.02)	−0.99 ± 0.74 (−1.39, −0.75)	1.14 ± 0.12 (1.07, 1.21)	27,069.56 ± 5843.59 (23,892.95, 30,246.17)	9.22 ± 3.45 (7.64, 11.10)	1.74 ± 1.02 (1.19, 2.29)
*p*-value	0.030 *	0.015 *	0.046 *	0.375	0.363	0.003 *	0.361

Notes: data are presented as mean ± standard deviation (95% confidence interval). AT MET, metabolic equivalent at the anaerobic threshold; Peak MET, peak metabolic equivalent during exercise testing; Peak VO2 Z-score, Z-score of peak oxygen consumption based on reference value [19]; RER, respiratory exchange ratio; Max CA Z-score, the maximum coronary artery Z-score of the right coronary artery and left anterior descending coronary artery based on Lambda-Mu-Sigma method [23]. * *p*-value < 0.05.

**Table 3:** Comparisons of cardiopulmonary fitness between sexes with normal and excessive BMI or FMI.

	AT MET	Peak MET	Peak VO2 Z-Score	Peak RER	PRPP	Pulse Oxygen	Max CA Z-Score
Total (normal + excessive body adipose)							
Boys (*N* = 61)	7.06 ± 1.25 (6.75, 7.37)	10.63 ± 1.76 (10.19, 11.07)	−0.53 ± 1.08 (−0.80, −0.26)	1.15 ± 0.08 (1.13, 1.17)	29,627.82 ± 6018.30 (28,117.51, 31,138.13)	9.67 ± 3.07 (8.90, 10.44)	1.24 ± 0.99 (0.99, 1.49)
Girls (*N* = 51)	6.38 ± 1.26 (6.03, 6.73)	9.20 ± 1.60 (8.76, 9.64)	−0.75 ± 0.66 (−0.93, −0.57)	1.16 ± 0.11 (1.13, 1.19)	27,595.00 ± 5856.42 (25,987.68, 29,202.32)	7.65 ± 2.63 (6.93, 8.37)	1.66 ± 0.98 (1.39, 1.93)
*p*-value	0.005 *	<0.001 *	0.189	0.590	0.074	<0.001 *	0.027 *
Normal BMI							
Boys (*N* = 40)	7.34 ± 1.09 (7.00, 7.68)	11.16 ± 1.54 (10.68, 11.64)	−0.48 ± 1.31 (−0.89, −0.07)	1.15 ± 0.08 (1.13, 1.17)	30,351.62 ± 6397.68 (28,368.96, 32,334.28)	10.39 ± 2.99 (9.46, 11.32)	0.88 ± 0.86 (0.61, 1.15)
Girls (*N* = 41)	6.42 ± 1.36 (6.00, 6.84)	9.20 ± 1.71 (8.68, 9.72)	−0.42 ± 0.52 (−0.58, −0.26)	1.16 ± 0.12 (1.12, 1.20)	30,092.50 ± 5946.32 (28,272.33, 31,912.67)	10.07 ± 3.43 (9.02, 11.12)	1.46 ± 0.79 (1.22, 1.70)
*p*-value	0.001 *	<0.001	0.788	0.660	0.851	0.655	0.002 *
Normal FMI							
Boys (*N* = 46)	7.30 ± 1.11 (7.08, 7.62)	11.10 ± 1.49 (10.67, 11.53)	−0.45 ± 0.58 (−0.62, −0.28)	1.15 ± 0.09 (1.12, 1.18)	31,532.55 ± 6760.50 (29,578.86, 33,486.24)	10.73 ± 3.42 (9.74, 11.72)	0.98 ± 0.75 (0.76, 1.20)
Girls (*N* = 38)	6.67 ± 1.12 (6.31, 7.03)	9.51 ± 1.52 (9.03, 9.99)	−0.64 ± 0.49 (−0.80, −0.48)	1.17 ± 0.10 (1.14, 1.20)	27,928.59 ± 5994.58 (26,022.59, 29,334.59)	6.92 ± 1.82 (6.34, 7.50)	1.46 ± 0.92 (1.17, 1.75)
*p*-value	0.012 *	<0.001 *	0.113	0.343	0.011 *	<0.001 *	0.012 *
Excessive BMI							
Boys (*N* = 21)	6.53 ± 1.38 (5.94, 7.12)	9.63 ± 1.74 (8.89, 10.37)	−0.56 ± 0.95 (−0.97, −0.15)	1.16 ± 0.09 (1.12, 1.20)	29,247.82 ± 5856.87 (26,742.80, 31,752.84)	9.29 ± 3.07 (8.08, 10.60)	1.43 ± 1.00 (1.00, 1.86)
Girls (*N* = 10)	6.20 ± 0.75 (5.74, 6.66)	9.19 ± 1.06 (8.53, 9.85)	−0.84 ± 0.85 (−1.37, −0.31)	1.15 ± 0.08 (1.10, 1.20)	26,985.85 ± 5742.53 (23,426.59, 30,545.11)	7.07 ± 2.05 (5.80, 8.04)	1.71 ± 1.03 (1.07, 2.35)
*p*-value	0.397	0.393	0.419	0.758	0.322	0.025 *	0.485
Excessive FMI							
Boys (*N* = 15)	5.69 ± 0.98 (5.19, 6.16)	8.4 ± 0.99 (7.90, 8.90)	−1.04 ± 1.30 (−1.70, −0.38)	1.16 ± 0.09 (1.11, 1.21)	28,910.07 ± 5452.82 (26,150.56, 31,669.58)	9.70 ± 2.92 (8.22, 11.18)	1.37 ± 1.04 (0.84, 1.90)
Girls (*N* = 13)	6.67 ± 1.12 (6.18, 7.28)	9.51 ± 1.52 (8.98, 10.34)	−0.64 ± 0.49 (−0.91, −0.37)	1.17 ± 0.10 (1.12, 1.22)	27,928.59 ± 5994.58 (24,669.90, 31,187.28)	6.92 ± 1.82 (5.93, 7.91)	1.46 ± 0.92 (0.96, 1.96)
*p*-value	0.022 *	0.036 *	0.284	0.785	0.656	0.005 *	0.810

Notes: data are presented as mean ± standard deviation (95% confidence interval). AT MET, metabolic equivalent at the anaerobic threshold; Peak MET, peak metabolic equivalent during exercise testing; Peak VO2 Z-score, Z-score of peak oxygen consumption based on reference value [19]; RER, respiratory exchange ratio; Max CA Z-score, the maximum coronary artery Z-score of the right coronary artery and left anterior descending coronary artery based on Lambda-Mu-Sigma method [23]. * *p*-value < 0.05.

## Data Availability

Individual participant data that underlie the results reported in this article after deidentification might be shared. Proposals should be directed to Gabbrile@vghks.gov.tw for application.
